# Longitudinal qualitative assessment of meaningful symptoms and relevance of WATCH-PD digital measures for people with early Parkinson’s

**DOI:** 10.1007/s00415-024-12789-0

**Published:** 2025-01-15

**Authors:** Jennifer R. Mammen, Aaron Lerner, Raunak Al-Rubayie, Melissa Kostrzebski, Diane Stephenson, Yuge Xiao, Chris Redmond, Mirinda Tyo, Varun G. Reddy, Peggy Auinger, E. Ray Dorsey, Jamie L. Adams

**Affiliations:** 1 College of Nursing and Health Sciences, University of Massachusetts, Dartmouth, Dartmouth USA; 2 Center for Health + Technology, University of Rochester Medical Center, Rochester, NY USA; 3 Department of Neurology, Medical Center, University of Rochester, Rochester, NY USA; 4 Critical Path Institute, Tucson, USA; 5 Michael J Fox Foundation for Parkinson’s Research, New York, USA; 6 Person With Parkinson’s Disease, New York, USA

**Keywords:** Parkinson’s, Qualitative, Symptoms, Patient experience, Digital health technology

## Abstract

**Background:**

Longitudinal qualitative data on what matters to people with Parkinson’s disease are lacking and needed to guide patient-centered clinical care and development of outcome measures.

**Objective:**

To evaluate change over time in symptoms, impacts, and relevance of digital measures to monitor disease progression in early Parkinson’s.

**Methods:**

In-depth, online symptom mapping interviews were conducted with 33 people with early Parkinson’s at baseline and 1 year later to evaluate (A) symptoms, (B) impacts, and (C) relevance of digital measures to monitor personally relevant symptoms. Maps and transcripts were coded for frequencies, Likert scale rankings (0 = not present to 4 = most bothersome), and thematic findings. Wilcoxon Signed Rank test was used to evaluate change over time.

**Results:**

Other than walking and balance, most motor symptoms did not change significantly from baseline to 1 year later. Multiple significant changes were observed in non-motor areas (cognition, speech, sleep, mood, fatigue, pain; *p* < 0.05) and functional impacts (mobility, effort to do usual activities, personal comfort; *p* < 0.05). Thematic analysis revealed ability to cope with and compensate for *actual* or *anticipated* symptoms reduced disruptions to well-being and changed how bothersome symptoms were. All digital measures targeted symptoms that were personally important to most participants (> 80%).

**Conclusion:**

Non-motor and walking/balance symptoms changed sooner than other motor symptoms during the course of 1 year. Evaluation of coping and compensatory mechanisms may be essential to understanding symptom bothersomeness at a given point in time. Smartphone and smartwatch digital measures were relevant to personally meaningful symptoms of early PD.

**Supplementary Information:**

The online version contains supplementary material available at 10.1007/s00415-024-12789-0.

## Background

A growing body of research has been conducted to identify the symptoms of Parkinson’s disease (PD) that matter most to people with Parkinson’s disease (PwP), yet understanding as to how priorities might change with time remains limited [1–4]. Systematic review of recent studies point to a range of motor and non-motor symptoms that are prevalent and bothersome in early stages [5], with important differences compared to later stages reported in cross-sectional studies [6, 7]. However, longitudinal assessment of symptoms and impacts to demonstrate within-person change over time is lacking. This information is greatly needed to inform priorities for clinical care and support development of new outcome measures that target meaningful aspects of health [8, 9].

At present, there are no outcome measures sufficiently sensitive to detect clinically meaningful change in early PD [10–12]. This has hindered clinical care and the development of new drugs to delay or halt disease progression [13, 14]. Thus, development of sensitive and reliable measures to monitor disease progression is a top priority that is contingent upon understanding what matters to people at different stages of disease [15]. This study aimed to systematically evaluate personally meaningful symptoms and impacts of disease longitudinally, along with perceived relevance of new digital monitoring technology to capture what mattered to people with Parkinson’s.

## Methods

### Study background

This longitudinal qualitative study was conducted as a companion study to the WATCH-PD2 (Wearable Assessments in The Clinic and at Home in PD) study [16]. WATCH-PD2 is an ongoing longitudinal, multi-center trial evaluating the capability of new digital measures to detect change over time in motor and cognitive function among individuals with early untreated PD (< 2 years from clinical diagnosis) versus healthy controls [17]. Participants were recruited from 17 Parkinson Study Group research sites. They were evaluated clinically and completed nine smartphone and smartwatch-based tasks daily, initially at 0, 1, 3, 6, 9, and 12 months in clinic and biweekly at home, and then for 1 week every 3 months thereafter. Digital measures included walking and balance tests, bilateral finger tapping, hand/shape rotation, recorded speech, and cognitive tasks (Supplement A).

This qualitative study began at the conclusion of Year 1 of the digital study to systematically assess (1) personally meaningful symptoms and impacts of Parkinson’s and (2) relevance of the WATCH-PD smartphone and smartwatch digital measures (hereafter digital measures) to monitor these meaningful aspects of disease. Baseline digital data and Year 1 qualitative data were reported previously [1, 17, 18]. This manuscript presents longitudinal qualitative findings, including self-percieved changes in PD symptoms and relevance of digital measures over time. 

### Setting, sample

**Fig. 1:**
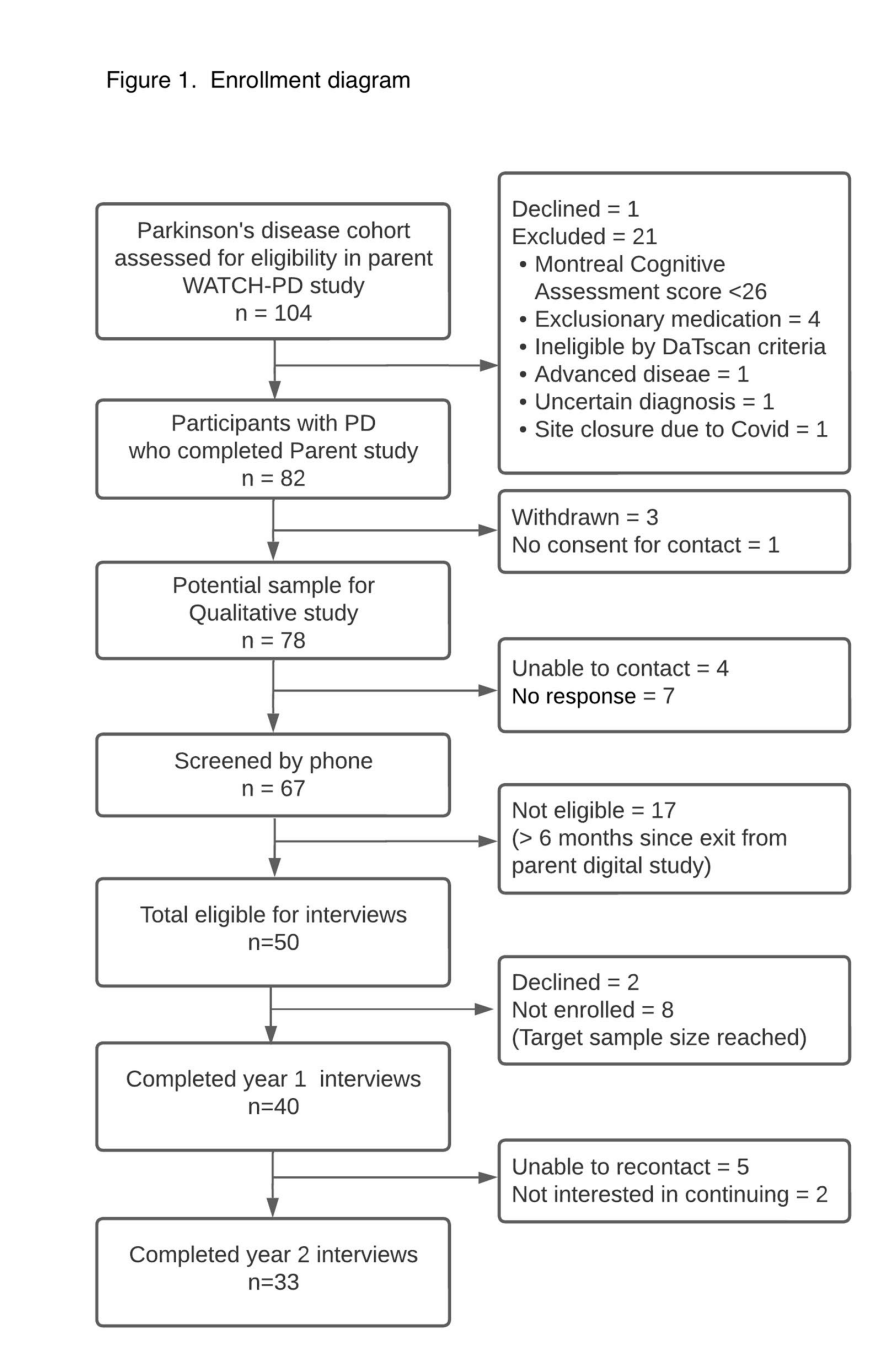
Enrollment diagram

All individuals from the PD cohort who completed the Year 1 WATCH-PD qualitative interviews (*N* = 40) were invited to complete a second interview 1 year later. Screening and enrollment statistics are presented in Fig. 1. Incentives were offered for participation.

### Data collection

Interview procedures were the same for both years. A concurrent mixed-methods design was used. Data collection began with a brief online survey of current PD symptoms and perceived relevance of nine digital measures, followed by a 2-hour online interview using (1) symptom mapping; (2) standardized cognitive debriefing on digital measures; and (3) mapping of digital measures to meaningful symptoms identified in the map.

*Survey* [19, 20]. Demographic data were obtained and reviewed with the participant for accuracy. Participants were asked about PD medication use, current symptoms, and personal relevance of digital measures (Supplement B). This was used as a starting point to inform the online interview.

*Online interviews.* The same interviewer conducted interviews at both time points (JRM; white, female, PhD prepared nurse practitioner) via Zoom video-conferencing. Sessions were recorded with participants’ permission. The interview followed a semi-structured interview protocol (Supplement C) and was conducted in three parts: (1) symptom mapping, (2) cognitive interviewing about digital measures, and (3) mapping of measures to personal symptoms to evaluate relevance. The interview protocol was developed by a group of experts, including people with lived experience and regulators, with attention to patient-focused drug-development guidance [18].

### Part 1: symptom mapping

**Fig. 2:**
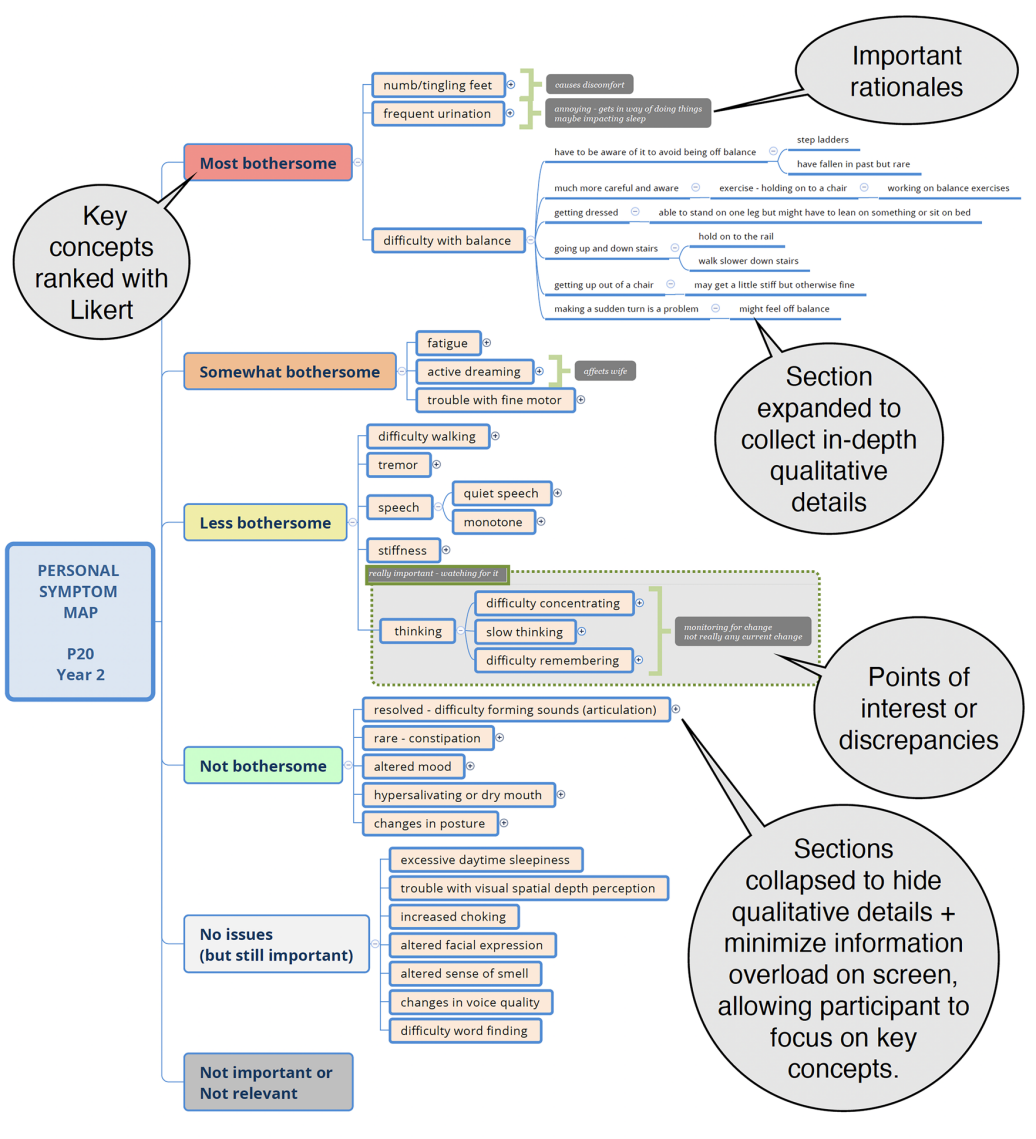
Symptom map schema

Symptom mapping is a hybrid Qual/Quant technique in which participants are assisted by a trained interviewer to create a visual word diagram of their personal symptom experiences using mind mapping software [18]. A symptom map uses branching logic to define key concepts (i.e., symptoms) within a single topic area (meaningful symptoms of PD) with qualitative details about the symptom experiences represented as concise summaries in the participants own words, attached to each key concept. At the conclusion of an interview, key concepts are sorted by the participant within a quantitative frame work, such as a Likert scale, to identify what matters from the individual’s perspective. Figure 2 illustrates this schema, and a full symptom map is shown in Supplement D.

The interviewer reviewed each participant’s survey data prior to the interview and entered all symptoms reported on the survey into the map as a starting point for the activity. Symptoms were recorded as yellow nodes, and dependent lines attached to nodes were used to represent impacts and any details about the symptom [21]. During the online interview, steps for building maps were: (1) entering spontaneously reported symptoms and related impacts believed to be attributable to PD; (2) probing for common symptoms or impacts not spontaneously mentioned; (3) rank ordering symptoms by how bothersome they are currently (range: most bothersome to not bothersome); (4) adding call out details that explain what makes symptoms more or less bothersome; (5) comparing the current map to the prior year’s map to explore changes over time. Steps 1, 2, 4, and 5 provide in-depth qualitative data on lived experiences (maps and transcripts), while Step 3 enabled quantification of symptoms based on the underlying Likert scale values of the bothersomeness framework. Probed symptoms were identified in prior literature reviews and from Year 1 interviews [1]. All symptom maps were co-created by the interviewer and participant, with iterative, real-time editing of maps and in-depth discussion used to ensured that the participant’s experience was accurately depicted (member checking).

### Part 2: cognitive interviewing

Following a brief break, the interviewer performed cognitive debriefing regarding the WATCH-PD digital measures. The interview protocol (Supplement B) included four standardized questions asked both years, plus further questions about relevance in Year 2 raised during analysis of Year 1 interviews. Standardized questions evaluated if participants understood what the task was asking them to do, what actions they used to complete it, what symptoms they believed it measured, and perception of relevance. Additional qualitative items in Year 2 asked participants to confirm if the symptom assessed by the digital measure was currently present, personally important (regardless of present), functionally limiting in any way, and personally bothersome (physically or psychologically). Finally, participants were asked if the digital measure was a relevant approach to assessing the symptom, if it related to real life for them, could be useful for monitoring change over time, and was personally relevant at their current stage of disease.

### Part 3: mapping tasks to personal symptoms

Lastly, participants returned to the symptom maps and were asked to integrate pictographs of each digital measure into their personal map next to the symptom(s) they felt the measure was relevant to, or to indicate if it was not relevant. This was done to confirm whether digital measures were associated with meaningful symptoms. The interview concluded with a final opportunity to review and edit the map. Participants were optionally given copies of their symptom maps at the conclusion of the interview.

### Data analysis

Content coding of maps and thematic analysis of narratives were conducted by JRM, AL, and RA as described in Supplement E [22]. Maps were coded in Excel for presence and bothersomeness of symptoms, impacts, and relevance of digital measures. Descriptive statistics were computed for frequencies of symptoms and impacts. Wilcoxon Signed Rank Test was used to assess for differences in relative bothersomeness of symptoms and relevance of digital measures (Likert scale range 0–4) and McNemar’s was computed for presence/absence of impacts from Year 1 to Year 2 using SPSS version 29.

### Rigor and validity

Measures to enhance validity included data triangulation (survey, map, interview), member-checking, peer-debriefing, use of two coders at each stage for validation, and a formal audit trail [23]. Representative quotes are presented with numeric identifiers.

## Results

### Sample and interview characteristics

Demographics are shown in Table 1. Participants were an average of 3.2 years since diagnosis, and most were taking Parkinson’s medication (e.g., carbidopa/levodopa, amantadine, rasagiline, pramipexole) at the time of the second interview (75.8% Year 2 vs. 40% at Year 1).

**Table 1:** Demographic characteristics

	Parent study (*n* = 82)	Year 1 interview *n* = 40	Year 2 interview *n* = 33
Age, years	63.3 (SD 9.4)	63.9 (SD 8.8)	65.92 (SD 8.9)
Female, *n* (%)	36 (43.9%)	19 (47.5%)	13 (41.9%)
Race/ethnicity, *n* (%)			
White	78 (95.1%)	37 (92.5%)	29 (93.5%)
Asian	3 (3.7%)	3 (7.5%)	2 (6.5%)
Not specified	1 (1.2%)	–	–
Hispanic or Latino, *n* (%)	3 (3.7%)	1 (2.5%)	0%
Education > 12 years, *n* (%)	78 (95.1%)	40 (100.0%)	33 (100%)
PD duration, years	0.8 (SD 0.6)	2.1 (SD 0.9)	3.2 (0.7)
Taking medications for PD, *n* (%)	–	16 (40.0%)	25 (75.8%)

### Symptom maps: comparison of most bothersome symptoms from Year 1 to Year 2

**Fig. 3:**
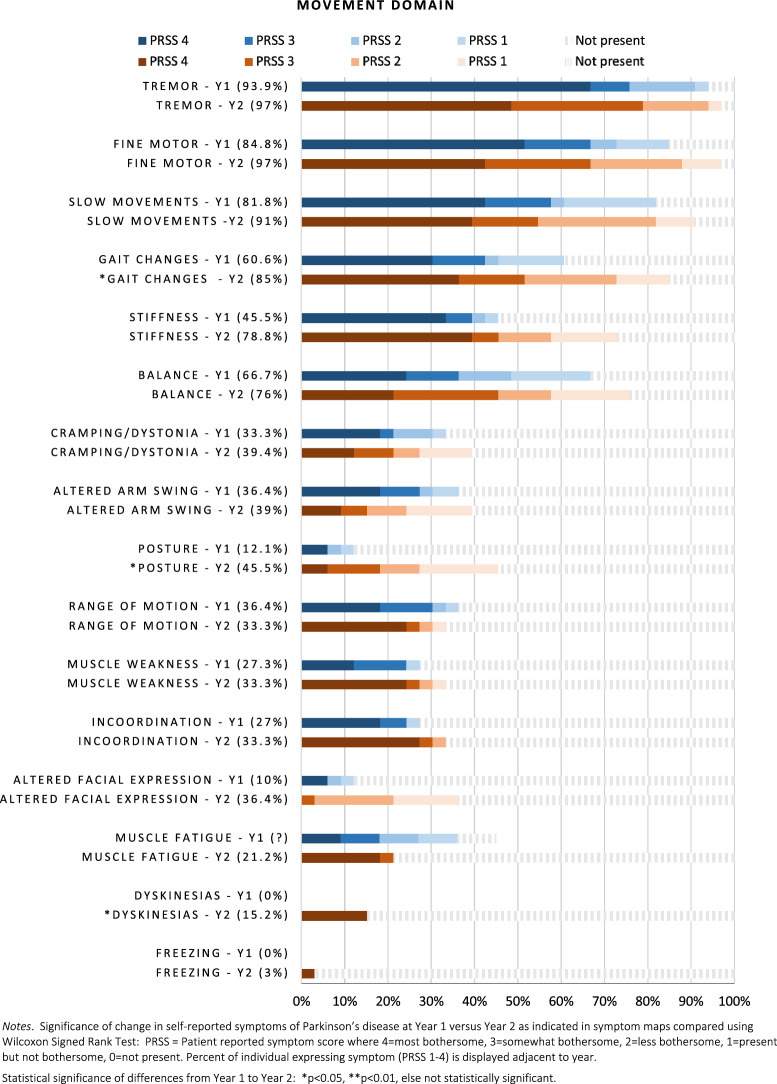
Movement symptoms

**Fig. 4:**
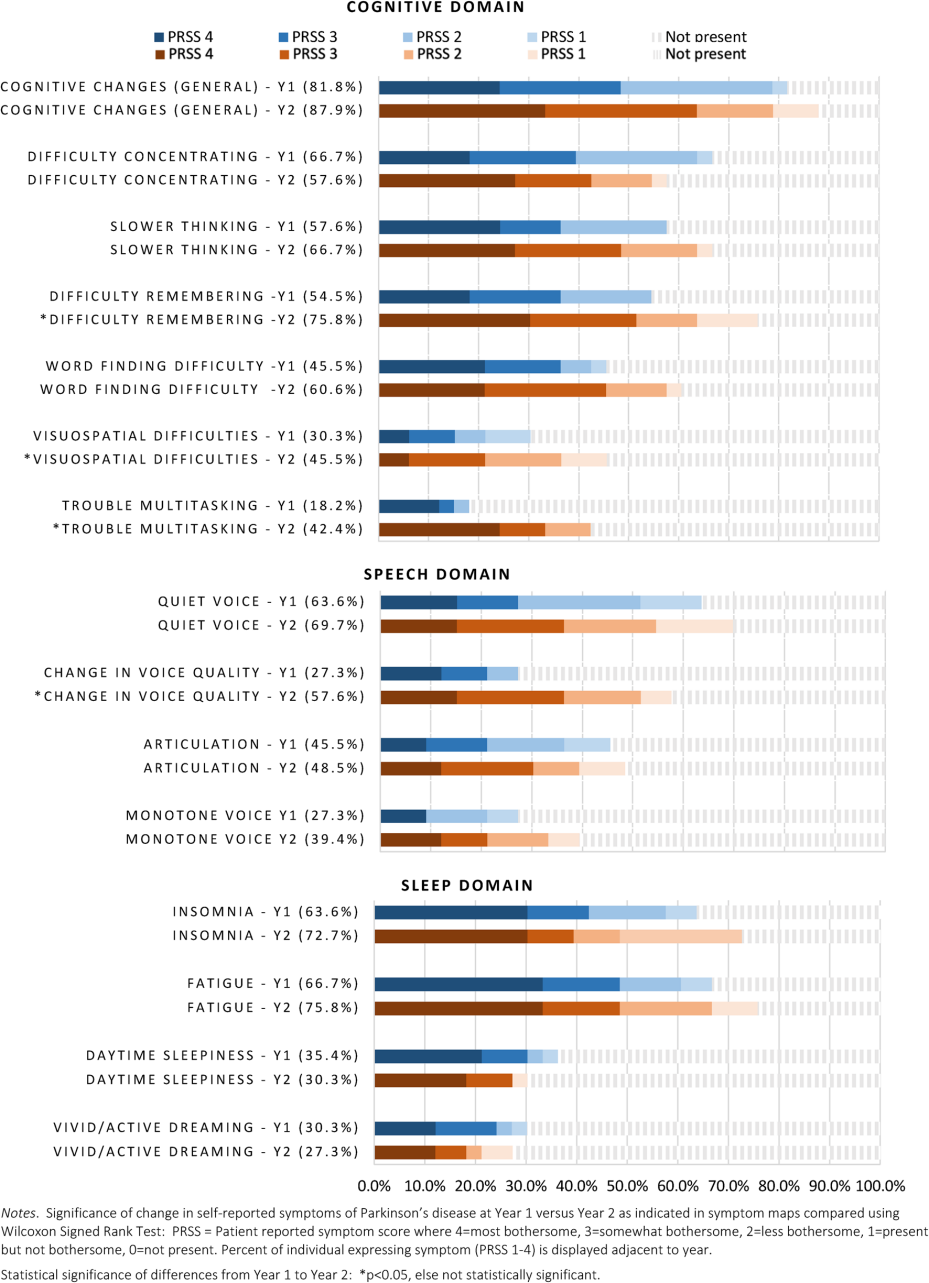
Cognitive speech and sleep symptoms

**Fig. 5:**
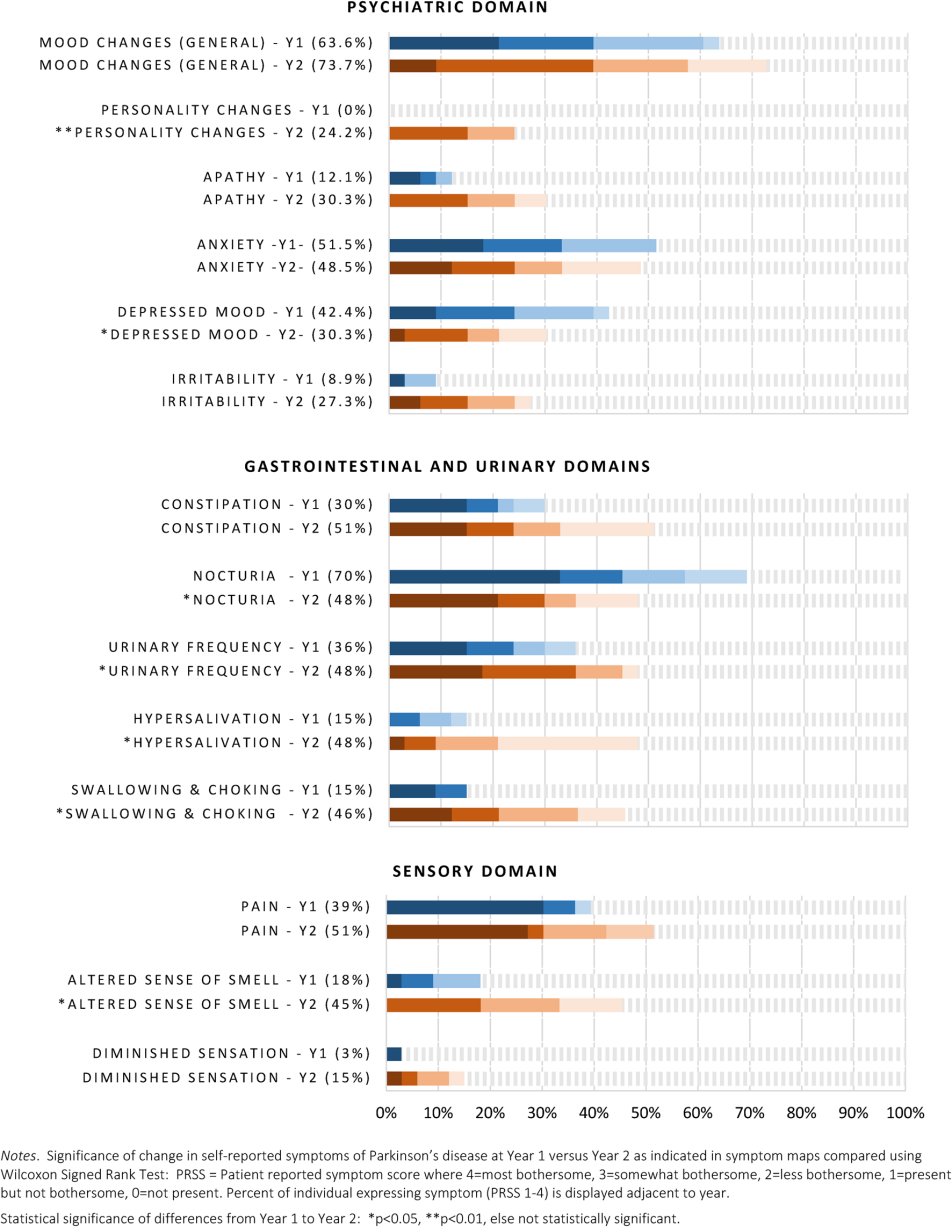
Psychiatric, genitourinary, and sexual symptoms

Longitudinal comparisons of symptom frequencies and relative bothersomeness are presented in Figs. 3, 4, 5. Top motor symptoms remained consistent at both time points: tremor, fine motor, slow movement, gait changes, stiffness and balance. Of these, only gait difficulties increased in prevalence (60% vs. 85%), and bothersomeness from Year 1 to Year 2 (median 1 = Not bothersome vs. 3 = somewhat bothersome; *Z* = −2.03, *p* ≤ 0.05). Although slightly more common, tremor and slow movements became somewhat less bothersome over time but did not achieve statistical significance. Posture changes also increased in frequency (12% vs. 45%) and became more bothersome (*Z* = −2.4, *p* ≤ 0.05). Dyskinesias, not previously present, were reported by 15% of the sample by Year 2.

Top non-motor symptoms for both years were cognitive, mood changes, insomnia, fatigue, and pain. There was a significant increase in trouble remembering and multi-tasking from Year 1 (*p* < 0.05). Changes in voice quality (i.e., raspy or different sound) also increased (27% to 57% prevalent; median rank 0 = not present vs. 1 = present/less bothersome at year 2, *p* < 0.05). Sleep symptoms remained constant. Personality changes were not noted in Year 1, but were reported by 24% of participants by Year 2 (*p* ≤ 0.01). Notably, participants reported *less* depression (42.4% vs. 33.3%) and fewer issues with nocturia (70% vs. 48%), although daytime urinary frequency was higher (36% Y1 vs. 48% Y2). Altered sense of smell and hypersalivation also increased in prevalence (18% vs. 45% and 15% vs. 48%, respectively) but were overall less bothersome.

### Comparison of physical and psychosocial impacts from Year 1 to Year 2

**Fig. 6:**
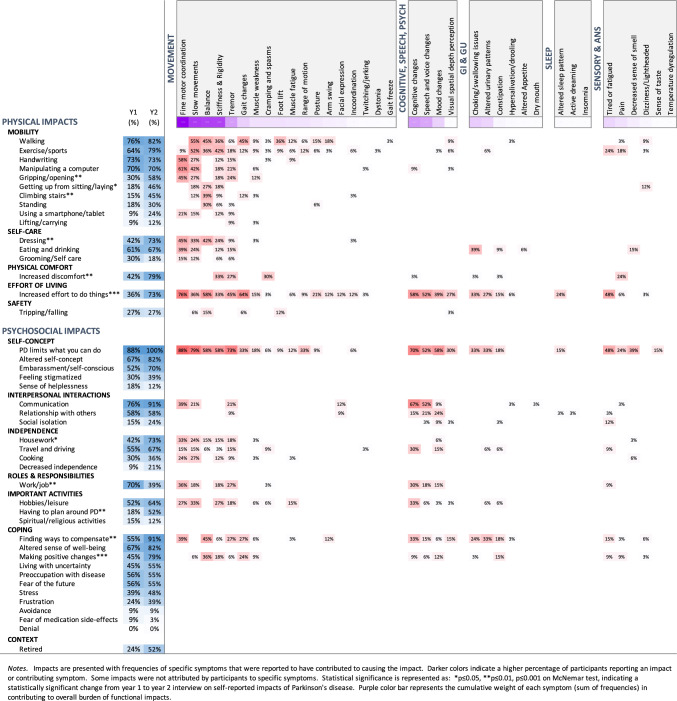
Impacts of symptoms

Fine motor difficulties, slow movements, impaired balance, and stiffness were the most reported causes of functional impairments. As shown in Fig. 6, by the second time point a year later, participants experienced significantly greater physical discomfort (42% vs. 79%, *p* ≤ 0.01) and effort to do usual activities (36% vs. 73%, *p* < 0.001). Impacts on physical functioning included altered mobility that most often affected walking (82%), exercise (79%), and tasks requiring hand dexterity (73% dressing, 73% handwriting, 70% computer use). Although less common, there were statistically significant increases in difficulty gripping and opening things, climbing stairs, and getting up from a sitting position.

Compared to Year 1, at Year 2 more participants reported that PD limited what they were able to do in various ways (88% vs. 100%), such as housework (42% vs. 73% *p* ≤ 0.05) and that their self-concept as a healthy, competent, and independent person was subsequently affected (67% vs. 82%). Greater difficulty was also experienced with interpersonal communication (91%, *p* ≤ 0.05). However, fewer people reported job related impacts (70% vs. 39%, *p* ≤ 0.05, possibly related to more individuals retiring (24% vs. 52%). There was also a notable and significant increase in use of compensatory behaviors (55% vs. 91%, *p* ≤ 0.01) and introduction of a range of positive changes to improve health, take control, and fight disease progression (e.g., exercise, meditation, healthy eating; 45% vs. 79%, *p* ≤ 0.001).

### Relevance of WATCH-PD digital measures to bothersome symptoms at Year 2

**Fig. 7:**
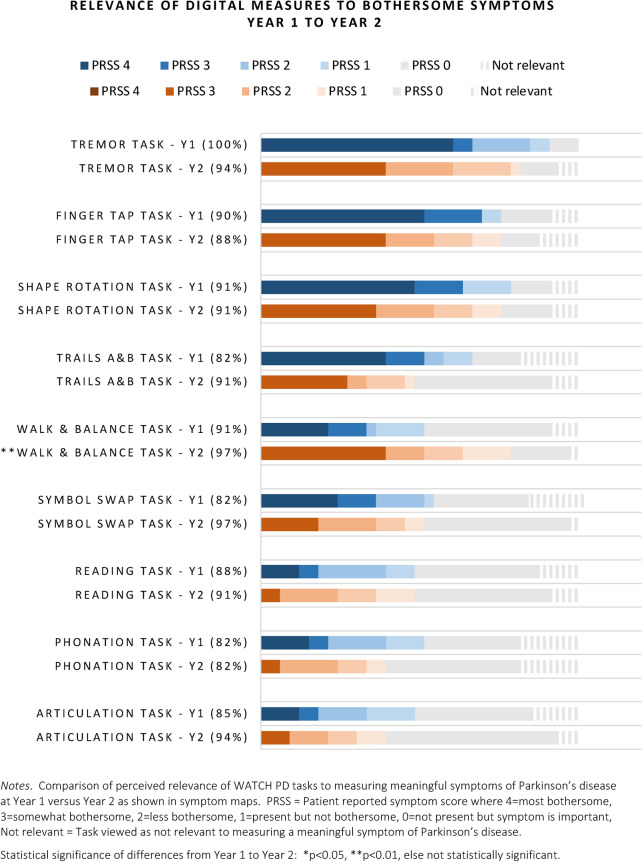
Relevance of digital measures as assessed by mapping

*Maps*. As shown in Fig. 7, all digital tasks were consistently relevant to more than 80% of participants. There was a significant gain in the reported relevance of the walking and balance task and the verbal symbol swap task (*p* < 0.01), corresponding with the statistically significant increases in these symptoms at Year 2.

*Cognitive interview*. For both years, excluding one instance, all participants understood what they were asked to do and generally completed tasks correctly (Column A, Table 2). A few participants reported using compensatory strategies to improve performance on certain tasks, such as turning the phone on the table during the shape-rotation task or using their tremor to inflate tapping scores on the finger-tapping task. These individuals recommended adding specific directions on what to do versus not do on potentially gameable measures to offset performance drive.

***P23****: It is easy to cheat on. Once you discover it, your body tells you, "I don't want to tap [correctly], I just want to do this [cheating]. It's easier to cheat than it is to actually do the thing.”*

***P21****: I could cheat… or even accidentally cheat. You are not really moving [the fingers] correctly, but it is still giving you some points. I could just tap them together rather than alternating.*

**Table 2:** Relevance of WATCH-PD tasks as assessed by cognitive interviewing during the Year 2 interview

WATCH-PD task	A	B	C	D	Quotes illustrating personal relevance
	Understood how to complete the task	Task measures personal PD symptoms	Task relates to things in real life	Task relevant to measure PD progression	
	n (%) [yes]	n (%) [yes]	n (%) [yes]	n (%) [yes]	
Movement					
Walking and balance (Y1)	33 (100%)	20 (60.6%)	32 (97%)	31 (94%)	P40: I really want to know if my walking gait is being impinged upon over time
					P3: Building a good database and a baseline is really important
Walking and balance (Y2)	33 (100%)	24 (72.7%)	27 (81.8%)	30 (90.9%)	P4: It would be interesting if my data changed … it probably has and I just am not even aware of it
					P24: It is not as relevant for me – I would want more time for the walking exercise
Resting tremor (Y1)	33 (100%)	28 (84.8%)	28 (84.8%)	32 (97%)	P4: Without knowing the data, I can't tell
Resting tremor (Y2)	33 (100%)	31 (93.9%)	17 (51.5%)	29 (87.9%)	P9: It is very relevant
					P1: It is the same test that my neurologist uses with me. If they think it is valid, I should too

Fine coordination					
Finger tapping (Y1)	32 (97%)	28 (84.8%)	25 (75.8%)	29 (87.9%)	P14: [It is] very relevant… I did this test that whole year, it got worse and worse each time I did it
					P21: I do not tap my fingers, but mov[ing] your fingers in a coordinated way is similar to things in life
Finger tapping (Y2)	33 (100%)	27 (81.8%)	17 (51.5%)	28 (84.8%)	P2: It is probably not a good measurement because I can game the system
Shape rotation (Y1)	33 (100%)	29 (87.9%)	27 (81.8%)	29 (87.9%)	P1: one issue with this test …if you have any fingernails at all, [it] does not work the way it should
					P36: I did not [rotate the phone] this time. I didn't realize I was cheating at the time
Shape rotation (Y2)	33 (100%)	28 (84.8%)	25 (75.8%)	29 (87.9%)	P16: I would call it dual-action fine motor. It is like using a Google Map to move the terrain from one spot to another. You need to have two different fingers on the touchpad

Speech					
Verbal reading (Y1)	33 (100%)	12 (36.4%)	28 (84.8%)	28 (84.8%)	P20: Being able to talk to other people, and have them understand you [is important]
Verbal reading (Y2)	33 (100%)	16 (48.5%)	26 (78.8%)	28 (84.8%)	P10: I think it is a good test because if I could not do it, I think it would signal I was progressing
Sustained phonation (Y1)	33 (100%)	15 (45.5%)	28 (84.8%)	26 (78.8%)	P25: it was measuring breath and how loud your vocal cords go… I do voice exercises saying, “Aah,”
Sustained phonation (Y2)	33 (100%)	16 (48.5%)	19 (57.6%)	23 (69.7%)	P17: We do not go, “Aaaaah.” None of our words are sustained like that. [but it is relevant] because the breathing part of it – that is one of my problems. Sometimes I feel like my diaphragm’s not full enough or I am not being able to push the air out
Verbal articulation (Y1)	33 (100%)	13 (39.4%)	25 (75.8%)	21 (63.6%)	P20: I think it is definitely relevant to me personally
					P30: It is a good way to test it, but they might come up with some variation to these syllables
Verbal articulation (Y2)	33 (100%)	16 (48.5%)	21 (63.6%)	27 (81.8%)	P11: I think it is a good phrase to [measure] deterioration over time. Maybe not so much comparing one patient to another, but within one patient I think it is a very useful measurement

Thinking					
Trails Aand B (Y1)	33 (100%)	20 (60.6%)	20 (60.6%)	29 (87.9%)	P25: I think I saw it more as a fine motor test, [but] you have got to understand the sequence of things
Trails A and B (Y2)	33 (100%)	12 (36.4%)	21 (63.6%)	29 (87.9%)	P16: It is measuring manual dexterity, mechanical and cognitive, that is why I think it is the most relevant of all the tests
Verbal aymbol swap (Y1)	33 (100%)	12 (36.4%)	29 (87.9%)	27 (81.8%)	P30: There is a glitch in the software… I am saying the number pretty fast, but the software is not getting it into the box as fast as I am saying it
Verbal symbol swap (Y2)	33 (100%)	15 (45.5%)	28 (84.8%)	31 (93.9%)	P11: As a bellwether of my overall cognition, it would be quite important. The fact that I donot currently have an issue with it [does not matter]. I think it is important

*N* = 33

**Fig. 8:**
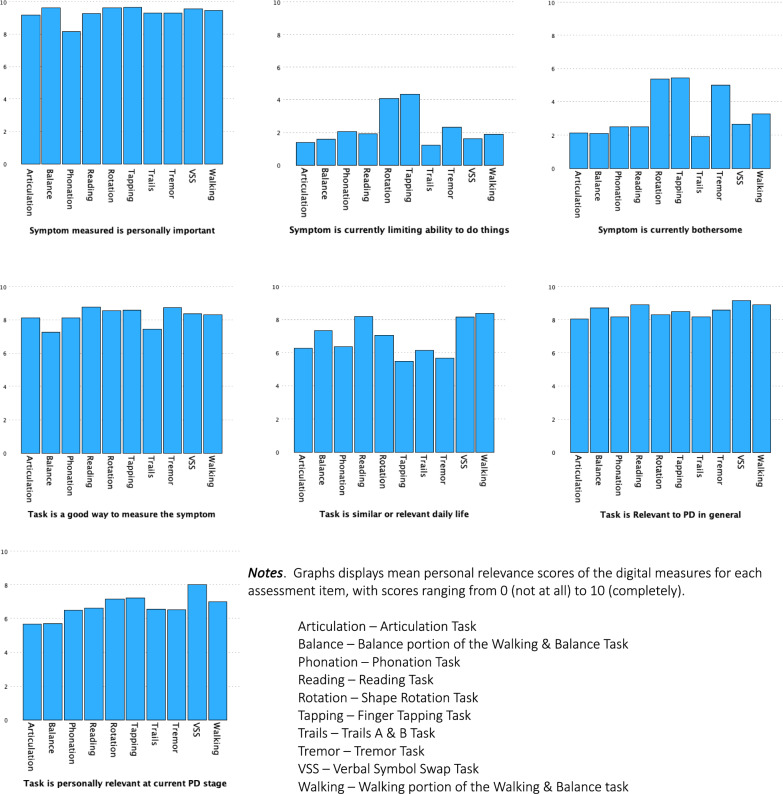
Relevance profiles of digital measures

Relevance profiles from cognitive interviewing for digital measures are shown in Fig. 8, with supporting statistics in Supplement F. On average, all measures targeted symptoms that were personally meaningful and were thus viewed as relevant to monitoring progression of PD symptoms (rated > 8/10). Nearly 75% of participants (25/33) suggested that making gait, balance, and cognitive tasks longer and “more challenging” would increase relevance of these measures to early PD, as the tasks seemed too easy to detect subtle, early changes. Common suggestions included standing on one leg to test balance, walking longer distances on uneven terrain, and performing activities while distracted (multi-tasking). Recommendations to address technological issues, including intermittent lack of responsiveness of the application to touch or voice, were reiterated at both years.

### Key theme: coping with PD is a highly variable and dynamic process

**Fig. 9:**
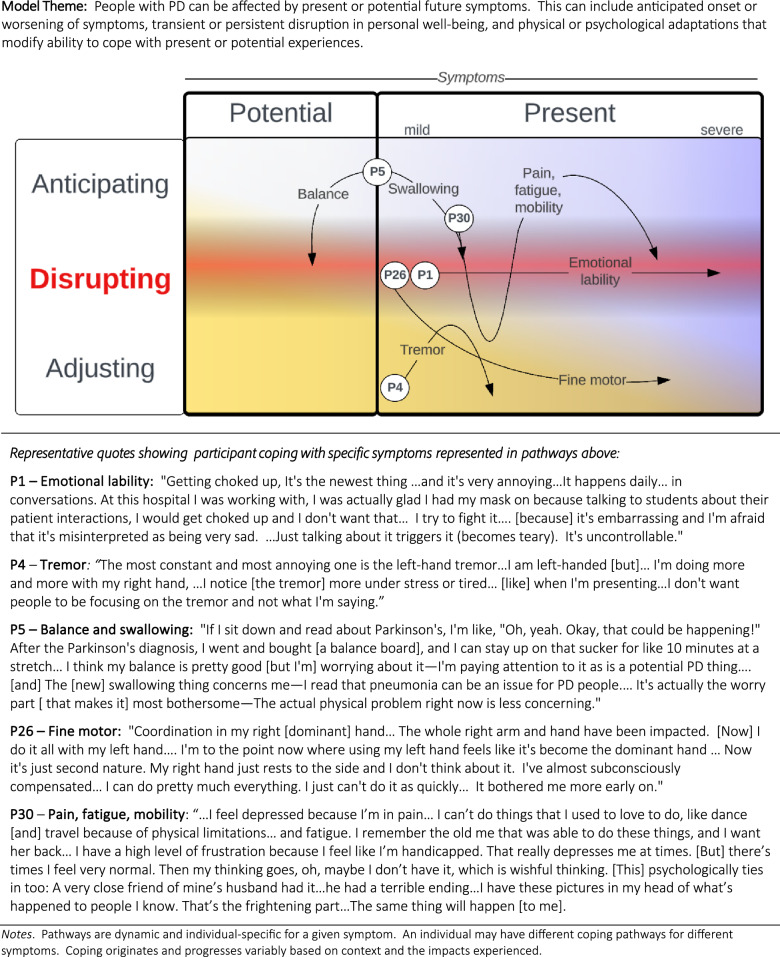
Model of coping with progressive disease

Participants experienced PD as a progressive disease and described trying to cope with both *present* and *potential* future symptoms (i.e., symptoms not yet experienced), as illustrated in Fig. 9. As one participant expressed: “*The reality is I am going to get worse”* (P4). Potential symptoms were those the individual worried they would develop later on, which was often the case with cognitive and speech symptoms (i.e., “Do I have it?” or “Will I get it?”). Both present and potential symptoms were a cause for worry about the future (i.e., “How bad will it get?”). In several instances, participants reported experiencing symptoms on survey that they later clarified in the interview were not actively present, rather something that the participant was worried about and “watching for.”

***P19****: I think I am more concerned about the future [symptoms].*

***P11****: This is the troublesome part about cognitive decline—I cannot tell. Is it happening?*

***P38****: The thing that bothers me is how bad is it going to get over time?*

Coping with PD varied within and between individuals and included: (1) anticipated onset/worsening of symptoms, (2) disruption in personal well-being, and (3) physical and/or psychologic adaptations (e.g., compensatory behaviors) that modified impact and improved ability to cope with present or potential experiences. In general, the extent to which a symptom was personally disruptive (i.e., “bothersome”) was influenced by the individual’s ability to physically accommodate or psychologically adjust. Situational context also affected the impact of the symptom (e.g., retirement vs. active employment). An individual might adapt successfully to one symptom yet experience disruption from others, based on context.

***P5****: Thinking is part of my work…I can work around slowness [but] I am not as sharp, and it concerns me for work.*

Physical accommodations included workarounds that provided a functional advantage in task performance. This included learning to perform activities of daily living (ADLs) with the non-affected hand (mousing, typing, grooming), avoiding certain types of clothing (buttons), and use of dictation to compensate for fine-motor difficulties with typing. Common cognitive adaptations included writing lists, use of memory aids and recall strategies, and setting reminders on phones to complete tasks.

***P2****: I am looking for other ways to get around the problem but still get things [done] the way I need to… I am always looking for shortcuts.*

Psychological adjustments included normalizing, meditation, positive thinking, reframing strategies, acceptance, and engagement in alternate positive activities such as support groups, exercise groups, or volunteer work (e.g., finding meaning).

***P36****: I am just used to it.*

***P20****: Parkinson’s is going to be with me…this [is] a life-changing thing, but I try to deal with it in a positive way.*

Importantly, the ability to mitigate the impact of a symptom by physical or psychologic adjustments resulted in a decreased tendency to report or prioritize the compensated symptom and translated to reduced perceived bothersomeness.

## Discussion

This is the first longitudinal qualitative study to systematically evaluate change over time in prevalence and bothersomeness of early PD symptoms and relevance of digital measures to monitoring what matters to patients. We found that the WATCH-PD digital measures were relevant to most people at both time points. Despite the ceiling effect, the consistently high ratings are encouraging and suggest that existing digital measures target symptoms that matter from the patient perspective. While not yet available for clinical practice due to being in early stages of validation, the hope is that phone or watch-based digital measures may eventually enable accessible, personalized disease monitoring that can aid in medication management, capitalizing on the widespread availability of these devices to support clinical care. However development of new measures will be needed, as many symptoms that were important to patients were not captured digitally.

We observed that baseline symptoms that were most bothersome (e.g., tremor, fine motor, slow movement, cognitive difficulties, mood changes, insomnia) remained a top priority one year later, which extends on knowledge from prior cross-sectional studies assessing symptoms at a single time point [1, 2, 4, 6]. Other than gait, most motor symptoms did *not* change significantly in 1 year. By contrast, worsening was observable in multiple non-motor areas, including cognitive, speech, psychiatric, sleep, and urinary domains. This has important implications for clinical trials, suggesting that trial duration of longer than 12 months might be needed to detect meaningful change in motor symptoms, whereas non-motor symptoms might have potential to show progression sooner. Clinicians may benefit by educating patients that Parkinson’s is more than a motor disease.

It is also important to note that the greatest change over 1 year was seen in functional impacts of symptoms on aspects of mobility, physical comfort, and increased effort to perform usual activities (e.g., work, housework, interactions with others). Based on participant narratives, we conclude that functional changes could serve as an earlier and potentially more sensitive indicator of early disease progression than individual symptoms alone. A possible explanation for this is that functional impairments can be attributable to multiple symptoms conjointly, such as altered fine motor, slowed movements, and tremor all impacting computer use. Thus, while change in a single symptom might not achieve statistically significant change in 12 months, functional impairments might become apparent sooner.

From a clinical standpoint, focusing on the *impact* of symptoms (i.e., what the person can/cannot do *or* now does differently) rather than the symptom itself may be a more accurate indicator of symptom burden, and align better with what matters to people living with PD. For example, despite increased prevalence and still being important, we found that certain motor symptoms (e.g., tremor, bradykinesia) were comparatively *less* bothersome a year later. Others have reported similar findings [6], however reasons for this have not been elucidated. One obvious explanation is the increased use of dopaminergic medication, which would alleviate symptoms of tremor and bradykinesia. However, our data also suggest that an individual’s ability to cope with and compensate for symptoms also moderates impact and bothersomeness. For instance, some participants indicated that mild-moderate tremor was easier to compensate for than cognitive symptoms, thus making the latter more bothersome to them.

Ability to compensate and cope appears to be a key factor in how meaningful a symptom is at any point in time. Lazarus and Folkman described coping as the “cognitive change and behavioral adaptation to something that exceeds the resources of the person” [24]. These concepts of coping have been explored across diverse diseases, including heart failure, chronic pain, diabetes, and other neurodegenerative diseases, with evidence of similar coping strategies [25–31]. In particular, the ability to cognitively restructure and problem solve has been identified as central to coping [32, 33], and in the present study reduced the burden of symptoms [34]. Related to this, we found a significant rise in positive behaviors to “take control” of PD, which appeared to improve quality of life [35]. In light of past and present findings, we would propose that assessing coping style and strategies may be helpful to understand symptom burden and disease progression [26].

Unfortunately, no consensus currently exists regarding what to measure with respect to coping in PD. Multiple models and frameworks have been proposed over the past 20 + years [25, 26, 36–39], which presents challenges for measurement. Harmonization of concepts will thus likely be needed to enable evaluation of coping in PD. Beyond clinical care, this is also relevant to drug trials, as failure to account for the potentially moderating impact of adaptive coping behaviors might result in misattribution of treatment efficacy or lack thereof.

Lastly, the conceptual model of coping presented in this manuscript points to anticipating and disrupting phases as important precursors to adjusting and adapting [1], which has broad relevance for both clinical practice and research. Anticipatory stress and grief have been extensively reported in other diseases [40] and are known to occur in later-stage PD [41]. Similarly, others have reported anticipation of worsening pain and posture changes [42, 43]. Combined with present findings, we conclude that anticipation of future symptoms and disease progression is almost certainly an important aspect of the early PD experience, and might result in substantial personal distress even in the setting of minimal symptoms. Thus, clinicians should aim to assess, educate, and intentionally support people with PD at all stages to achieve optimal coping, a new normal, and a preserved sense of self [44].

## Limitations

Limitations of this study include a smaller sample size, which is consistent with in-depth qualitative research, and representation of mostly white participants, attributable to parent study demographics. Greater diversity is needed in PD research and additional work will be needed to understand if findings reported here apply to non-white populations or those outside the US. It is possible that people with different characteristics (race, age, socioeconomic, or professional status) may prioritize symptoms differently. Furthermore, longer follow-up periods might be needed to evaluate change, as only modest worsening in symptoms was noted over 1 year. We believe this is useful information that can help to inform duration of future clinical trials. Lastly, reporting of symptoms was based entirely on the participant’s perceptions of what was or was not attributable to their PD. This could differ from clinical perspectives or be influenced by personal, cultural, or educational factors. Despite these noted limitations, our data clearly indicate what matters to people with PD and can help to guide patient-focused drug-development efforts and clinical care.

## Conclusion

This study emphasizes the importance of mixed qualitative and quantitative strategies to understand what matters to people with PD to guide patient-centric care and advance clinically meaningful endpoints. All digital measures were broadly relevant, but additional measures are needed that capture other important aspects of disease. Fewer changes were found in self-reported prevalence or bothersomeness of motor symptoms, whereas greater change was observed in non-motor areas and functional impacts. Bothersomeness of symptoms was affected by ability to cope (i.e., physically or psychologically adjust). Thus, evaluating disease progression via self-reported symptoms might require concurrent assessment of compensatory coping strategies to control for sources of variability that could otherwise obscure ability to detect change over time. Lastly, greater psychosocial support for individuals in the misnomered PD “honeymoon period” might be necessary to promote adjustment and alleviate distress [45].

## Supplementary Information

Below is the link to the electronic supplementary material.

Supplementary file1 Supplement A. WATCH-PD digital measures for Year 2 (PDF 158 KB)

Supplementary file2 Supplement B. Pre-interview Survey (PDF 82 KB)

Supplementary file3 Supplement C. Interview protocol for Year 2 (PDF 534 KB)

Supplementary file4 Supplement D. Sample symptom map. (PDF 742 KB)

Supplementary file5 Supplement E. Coding approach (PDF 37 KB)

Supplementary file6 Supplement F. Supporting data for relevance ratings of WATCH-PD digital measures (PDF 70 KB)

Supplementary file7 Supplement G. All symptom frequencies. (PDF 132 KB)

## Data Availability

De-identified datasets are available upon request.

## Declarations

### Quality in qualitative research statement

This study used COREQ criteria to guide reporting of qualitative findings.
