# Supplementary material for: Longitudinal qualitative assessment of meaningful symptoms and relevance of WATCH-PD digital measures for people with early Parkinson’s
Source: J Neurol. 2025 Jan 15;272(2):114. doi: 10.1007/s00415-024-12789-0 (PMC11735495; doi:10.1007/s00415-024-12789-0)
Supplement: Supplementary file 1 — Supplementary file1 Supplement A. WATCH-PD digital measures for Year 2 (PDF 158 KB) [file 415_2024_12789_MOESM1_ESM.pdf]

Supplement A. WATCH-PD digital measures evaluated for relevance from the patient perspective in Year 2

	TASK NAME	DOMAIN MEASURED	ACTIONS REQUIRED TO PERFORM ASSESSMENT	PICTOGRAPH
Smartwatch	Walking & Balance	Gait/balance	(1) Participant walks straight line for 1 minute.<br>(2) Participant stands with arms at sides for 30 seconds.	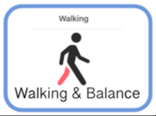
	Tremor Task	Tremor	(1) Participant rests hands in lap for 10 seconds.<br>(2) Participant extends arms out in front for 10 seconds.	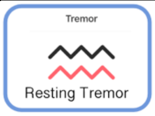
Smartphone Application	Finger Tapping	Fine motor	Participant performs rapid alternating finger movements by tapping two side-by-side targets with index and middle fingers.	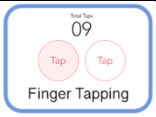
	Shape Rotation	Fine motor	Participant uses 1-2 fingers to move and rotate a pink object into the object outline as quickly as possible.	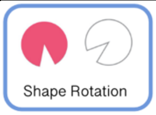
	Verbal Articulation	Speech	Participant repeats the syllables "pa ta ka" for 15 seconds.	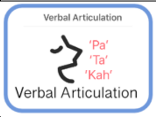
	Visual Reading	Speech	Participant reads a series of sentences printed on the screen.	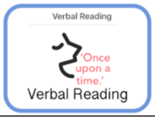
	Sustained Phonation	Speech	Participants performs sustained phonation task for 15 seconds.	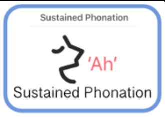
	Digit Symbol Substitution	Thinking	Participant is presented with a symbol and must speak aloud the corresponding number from a key.	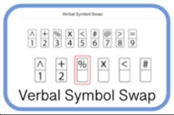
	Visuo-Spatial Working Memory	Thinking	REMOVED Y2: Participant is briefly shown four different colored boxes followed by a single, colored box and must indicate if the single box was in the previous set of four.	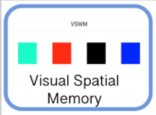
	Trail Making Task	Thinking	Participant must trace a set of alpha-numeric dots as quickly and accurately as possible using the index finger of the dominant hand.	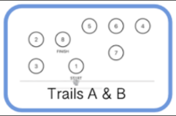

Notes. BrainBaseline application screenshots reprinted with permission from Clinical ink.
Supplement showing digital measures previously published in Journal of Parkinson's disease doi: 10.3233/JPD-225122 and reprinted with permission.
