# Supplementary material for: Longitudinal qualitative assessment of meaningful symptoms and relevance of WATCH-PD digital measures for people with early Parkinson’s
Source: J Neurol. 2025 Jan 15;272(2):114. doi: 10.1007/s00415-024-12789-0 (PMC11735495; doi:10.1007/s00415-024-12789-0)
Supplement: Supplementary file 2 — Supplementary file2 Supplement B. Pre-interview Survey (PDF 82 KB) [file 415_2024_12789_MOESM2_ESM.pdf]

WATCH-PD QUALITATIVE SUBSTUDY – SURVEY QUESTIONS

Note: *The following survey questions will be administered to* *<u>all</u>* *participants the WATCH-PD qualitative study. The purpose of these questions is data triangulation. Quantitative findings from the survey of all participants will provide support for the qualitative analysis and will enhance validity and transferability/generalizability of subsequent findings.*

This is a computer-based survey, which will be administered using Redcap.

Prior to administering the survey, the RA will confirm:

Participant has been deemed eligible	☐ Yes ☐ No
Participant has consented	☐ Yes ☐ No

Introduction to Survey

The purpose of this survey is to understand **<u>what symptoms of Parkinson's are most bothersome and important to you</u>** and how information captured in the WATCH-PD study relates to your experience with Parkinson's disease symptoms. This is a follow up study to the one you completed last year and will cover the same basic content to see if anything has changed over time.

The data we collect will be used to improve the use of digital health technologies to monitor important features of Parkinson's disease.

<u>We want to hear your perspectives</u>, so answer questions in whatever way you feel best reflects you.

Thank you for taking time to complete this survey!

Background

[Demographic data here – link to baseline data via Parent Study ID]

1. Please list any medications that you are currently taking for your Parkinson's disease

2. Are any of these new medications that you started since the last time you did this study in November 2021?

   (Yes/No)

3. Please list any new medications you are taking to help treat your Parkinson's Disease symptoms:

   (Open response – branching from Q1)

Section 1. Explore Parkinson's Disease Symptoms

1. Please describe the symptoms of Parkinson's disease that you experience in detail.

   (Open response)

2. Of the symptoms you described, **which are the most bothersome to you currently** and why?

   (Open response)

3. Have the symptoms you find most bothersome changed since the last time you completed this survey? If so, how?

   (Open response)

Of the following symptoms that were measured by WATCH-PD, please rate how bothersome each symptom is to you on a scale of 0-10, with 10 being the most bothersome.

[Scale changed to numeric 0-10 based upon participant feed back for ease of use.]

4. Shaking (tremors)

   (Range: 0=Not bothersome…10=Very bothersome)

5. Slow movements

   (Range: 0=Not bothersome…10=Very bothersome)

6. Difficulty walking

   (Range: 0=Not bothersome…10=Very bothersome)

7. Difficulty with balance

   (Range: 0=Not bothersome…10=Very bothersome)

8. Trouble with fine motor (example: buttoning your clothing)

   (Range: 0=Not bothersome…10=Very bothersome)

9. Altered mood (depressed, anxious, hopeless)

   (Range: 0=Not bothersome…10=Very bothersome)

10. Excessive sleepiness

    (Range: 0=Not bothersome…10=Very bothersome)

11. Feeling tired or fatigued

    (Range: 0=Not bothersome…10=Very bothersome)

12. Difficulty concentrating

    (Range: 0=Not bothersome…10=Very bothersome)

13. Difficulty remembering

    (Range: 0=Not bothersome…10=Very bothersome)

14. Slow thinking

    (Range: 0=Not bothersome…10=Very bothersome)

15. Difficulty forming sounds (articulating)

    (Range: 0=Not bothersome…10=Very bothersome)

16. Quiet speech

    (Range: 0=Not bothersome…10=Very bothersome)

17. Monotone speech

    (Range: 0=Not bothersome…10=Very bothersome)

18. Trouble with visual/spatial or depth perception (example: estimating distances)

    (Range: 0=Not bothersome…10=Very bothersome)

19. Of all these you have reviewed or reported, which symptoms are the <u>most important</u> to you overall?

    Open response

FINAL QUESTIONS

1. Do you have any comments or suggestions for how we could improve the use of digital devices to measure symptoms of Parkinson's disease that would be meaningful to you personally?

    (open response)

Conclusion

This concludes the survey. Thank you for your time and for all the insightful information and experiences you have shared!
