# Supplementary material for: Longitudinal qualitative assessment of meaningful symptoms and relevance of WATCH-PD digital measures for people with early Parkinson’s
Source: J Neurol. 2025 Jan 15;272(2):114. doi: 10.1007/s00415-024-12789-0 (PMC11735495; doi:10.1007/s00415-024-12789-0)
Supplement: Supplementary file 3 — Supplementary file3 Supplement C. Interview protocol for Year 2 (PDF 534 KB) [file 415_2024_12789_MOESM3_ESM.pdf]

PARTICIPANT INTERVIEW GUIDE INSTRUCTIONS AND QUESTIONS

Note: *Below is a semi-structured interview guide. It is to be used as a guide only. The actual areas of conversation are fluid and may be discussed at moments different from the order appearing below. The interview will be approximately 120 minutes. The interviewer may adapt the guide in order to cover the topics in the amount of time allotted for the session or in order to best elicit concepts from the participants.*

Prior to the start of the discussion, please check off:

Participant has been deemed eligible	☐ Yes ☐ No
Participant has consented prior to the discussion	☐ Yes ☐ No

Notes to Interviewer:

- This interview guide is meant to help guide the discussion, but not to be used as a verbatim script; probes and questions may change slightly depending on individual feedback.
- Additional unscripted probes to be used to gain further information or clarification may include:
  - **Clarification:** I don't quite understand that.
  - **Expressing understanding:** How did you cope with that?
  - **Justification:** Can you tell me a little bit more about why you chose that for your answer?
  - **Importance:** How important is this for you?
  - **Relationship:** I'm not sure how these 2 things are linked.
  - **Extending narrative:** Tell me a bit more about that.
  - **Accuracy:** Let's see if I've got that right.

Key for Interviewer:

- Questions/text to be asked of the participant
- *Notes to the interviewer (Do not read to participant)*

Introduction

Thank you for taking the time to speak with me today. Before we can start with the interview let's go over the information for the study.

We are talking to people such as yourself who have been participants in the WATCH-PD study. The purpose of our conversation today is to better understand how the data captured in the WATCH-PD study relates to your experience with Parkinson's disease symptoms and impacts. The interview data we collect will be used to support the use of wearable devices or digital health technologies that can record important features of Parkinson's disease progression in future clinical trials.

There are no treatments being tested in this study.

You are being asked to take part in one interview, which will be audio/video recorded to ensure we capture everything you say accurately. The interview will take about 120 minutes to complete. The recording will be transcribed and no names will appear in the written transcript. All your responses will be anonymous; your name will not be linked with any of your responses. Recording the interview is a required part of the study. If you do not want to be recorded, you may not take part in the study.

Your participation is voluntary, which means that you do not have to take part in the interview. You can skip any question you do not want to answer, and you can choose to stop the interview at any time. You will be compensated $X in the form of a gift card for your time after the interview.

Before we proceed, do you have any questions?

Address all questions the participant has before proceeding.

Before we get started, I would like to reiterate that this session will be audio recorded. However, your name will not be linked with the recording, transcription, or your responses during the interview.

Is it okay for me to record the conversation today?

If yes, continue to "Background for All Interviews."

If no: Unfortunately, since you do not agree to the recording of this session, you won't be able to participate in this study. Thank you for your willingness to consider participation in this study.

Background for All Interviews

- My role here is to ask questions and to listen. I will also be summarizing information at times. I will ask questions related to your experience and I will move the discussion from one question to the next to try to keep us on track so that we can finish on-time.
- I am not your medical doctor, so I am not qualified to give medical advice. I encourage you to follow-up with your regular doctor if you have any questions about your condition after this interview.
- Please feel free to let me know if you need a break. You can ask me questions at any time.

Any questions before we begin?

Begin Recorder: This is participant ID *[insert ID number here]* for the WATCH-PD Qualitative Sub Study on *[Date]*. Do I have your permission to record this interview? ***Verbal response required*****.** And can you please confirm that you read and signed the Informed Consent Form? ***Verbal response required*****.**

Section 1. Personally Important Parkinson's Disease Symptoms

The purpose of our conversation today is to better understand how the data captured in the WATCH-PD study relates to your experience with Parkinson's disease symptoms and impacts. As part of this I'm going to ask you to describe your symptoms of Parkinson's and then I will create a map or a "picture" of symptoms that are important to you, based upon what you tell me. As I create the map, please point out anything you see that needs adjusting, as this will help me to best represent your experience.

1. First, would you tell me what Parkinson's disease-related symptoms you currently experience? From your responses on the survey, I see that you listed….

   [Step 1 of the symptom mapping activity begins here. As the participant directs, the interviewer will map the participants symptoms by order of personal importance.]

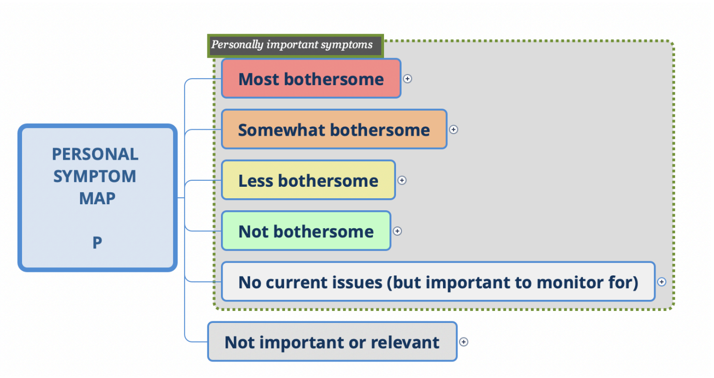

   - Probe: Are there other symptoms that you currently experience that you have not told me about and that I don't have in your map?
   - Probe: Looking at your previous map, I see you also listed [any symptoms not mentioned].
   - Probe [*yes/no*] on any they do not spontaneously state (e.g. tremor, slow movements, gait disturbances, fine motor coordination, speech articulation, cognitive impairment, daytime sleepiness, mood symptoms).
   - Probe: Are there any other symptoms that other people around you have observed you have that you haven't personally noticed?

2. Of those symptoms you mentioned, explain to me which are the most bothersome to you. What specifically makes those symptoms bothersome, and in what situations?

   [The interviewer will add concise details to the symptom map delineating what makes specific symptoms important/bothersome.]

3. Explain to me which symptoms are most important to you. Is that the same or different from what is most bothersome?
   - Probe: Have we captured all the symptoms that you experience correctly? Is there anything we missed?

4. Have your symptoms changed since the last time? If so, how have they changed and what do you think has contributed to change (improvement, worsening, etc.) – examples lifestyle, medication use, progression.
   - Probe: Allow participant to compare and reflect on current map vs. prior map

Section 2. Patient Perspectives on WATCH-PD Battery – Task Debriefing

In the WATCH-PD study you completed a range of tasks at home through use of the iPhone and Apple Watch and in the clinic. Now we will discuss those tasks.

The goal of the following questions is to determine if the tasks (and specifically what tasks) assessed via the WATCH-PD technologies are important/relevant/meaningful to patients. How do the tasks relate to how they feel and function?

<u>Ask each of the following standardized questions exactly as stated for each of the 10 tasks on the following page and record answers.</u>

A. You completed a **[Task name here]**. The screen looked like this:

Insert Task Image

1) (In your own words, describe what you think you were supposed to do in order to complete this task.) - asked at baseline and optionally repeated at Y2 and Y3 only if indicated.
2) In your own words, briefly describe what you actually did in order to complete this task. *(Describe)*
3) What symptom of Parkinson's did you think that this task was measuring? *(Describe)*
4) Have you personally experienced the symptom you feel the task is measuring? (*Never, past, present*)
5) How important is that symptom to you personally? *(Describe and rate 0-10*, ***regardless of present**)
6) How much do you feel this symptom has limited your ability to do things? *(Describe and rate 0-10)*
7) How bothersome has this symptom been for you? *(Describe and rate 0-10, ****regardless of limiting***)*
8) Do you feel like the task you completed is a good test of that symptom? *(Describe and rate 0-10)*
9) How is this task similar or relevant to things you do in your daily life?
10) How relevant do you feel this task is for measuring the progression of Parkinson's disease <u>in general</u>?
11) How relevant is this task to you <u>personally</u> for monitoring the PD symptoms that are important to you? *(Describe and rate 0-10)*
12) Are there any possible risks or disadvantages to you personally in participating in this task/assessment?

	TASK NAME	DOMAIN MEASURED	ACTIONS TO COMPLETE TASK	PICTOGRAPH FOR MAP
Smartwatch	Walking & Balance	Gait/balance	(1) Participant walks straight line for 1 minute.<br>(2) Participant stands with arms at sides for 30 seconds.	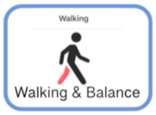
	Tremor Task	Tremor	(1) Participant rests hands in lap for 10 seconds.<br>(2) Participant extends arms out in front for 10 seconds.	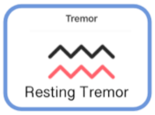
Smartphone Application	Finger Tapping	Fine motor	Participant performs rapid alternating finger movements by tapping two side-by-side targets using index and middle fingers.	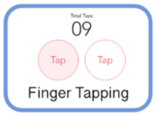
	Shape Rotation	Fine motor	Participant uses 1-2 fingers to move and rotate a pink object into the object outline as quickly as possible.	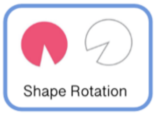
	Verbal Articulation	Speech	Participants performs sustained phonation task 15 seconds.	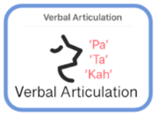
	Visual Reading	Speech	Participants reads a series of sentences printed on the screen.	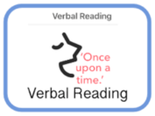
	Sustained Phonation	Speech	Participants repeats the syllables "pa ta ka," for 15 seconds.	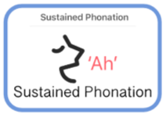
	Digit Symbol Substitution	Thinking	Participant is presented with a symbol and must speak aloud the corresponding number from a key.	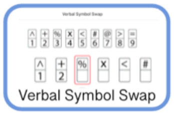
	Visuo-Spatial Working Memory	Thinking	REMOVED IN YEAR 2: Participant is briefly shown four different colored boxes followed by a single, colored box and must indicate if the single box was in the previous set of four.	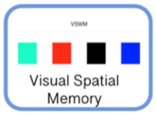
	Trail Making Task	Thinking	Participant must trace a set of alpha-numeric dots as quickly and accurately as possible using the index finger of the dominant hand.	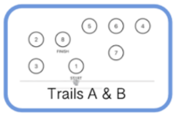

Section 2A: Mapping Activity (Relating Tasks to Personal Symptoms)

Now let's incorporate each of the WATCH-PD tasks we just talked about into your personal symptom map where you feel they fit best. (USE PICTOGRAPHS - One task per card).

1. Where would you place each task card in relationship to the symptoms in your personal symptom map?
   - Probe: clarify what makes task relevant or not

[As the participant directs, the interviewer will integrate the WATCH-PD tasks into the personal symptom map, relating each task to meaningful symptoms, or indicating if the task is not relevant to the participant, along with details as to what makes those tasks relevant or not relevant.]

Closing questions

1. In general, how do you feel about using digital technologies for routine ongoing monitoring of PD symptoms?
   - Probe: what do you see as the benefits of ongoing monitoring (personal/general)?
   - Probe: what do you see as the disadvantages of ongoing monitoring?
2. If you were choosing:
   a. When would you want to start monitoring for different PD symptoms? (e.g., before symptoms start, after symptoms are present, only when they become bothersome…)
   - Would you want to monitor for symptoms you do NOT currently experience?

   b. How often would you want to monitor PD symptoms? (e.g. continuous, daily, weekly, monthly)
   c. Would you want to get feedback on your results?
   - Would you want to be told if the monitoring showed your symptoms were getting worse?

   d. Would you like the option to be more involved in the research process? If so how?
3. What was your experience with or perception of the symptom mapping process?
   a. Are there any changes you think we should make to this technique?
   b. What did you like or dislike about it?
4. Do you have any suggestions about ways we could improve our assessment of personal meaningfulness of the WATCH-PD tasks? (i.e. questions asked, visualization approaches, or relating back to the personal symptom map)
   a. Are there any changes you think we should make to this technique?
   b. What did you like or dislike about it?
5. Do you have any other thoughts or comments?

Thank you for your time and for all the insightful information and experiences you have shared with me today. Now, let’s discuss the next steps before we end the interview.

[Stop recording and go through any closing logistical items with the participant.]
