# Supplementary figures and images for: Longitudinal qualitative assessment of meaningful symptoms and relevance of WATCH-PD digital measures for people with early Parkinson’s
Source: J Neurol. 2025 Jan 15;272(2):114. doi: 10.1007/s00415-024-12789-0 (PMC11735495; doi:10.1007/s00415-024-12789-0)

Supplement D. Sample symptom map from Year 2 WATCH-PD interviews

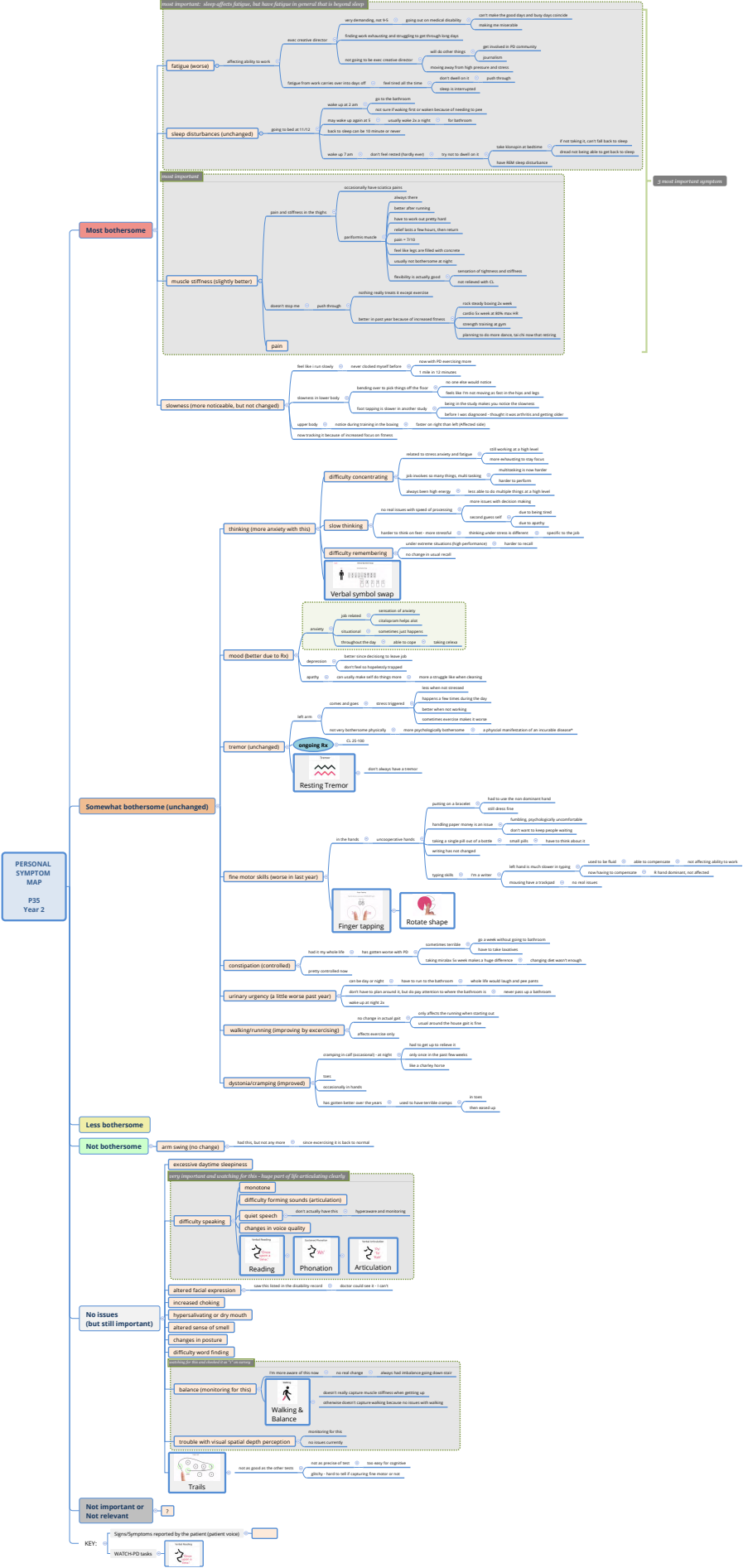

Supplement: Supplementary file 4 — Supplementary file4 Supplement D. Sample symptom map. (PDF 742 KB) [file 415_2024_12789_MOESM4_ESM.pdf]
