# Supplementary material for: Longitudinal qualitative assessment of meaningful symptoms and relevance of WATCH-PD digital measures for people with early Parkinson’s
Source: J Neurol. 2025 Jan 15;272(2):114. doi: 10.1007/s00415-024-12789-0 (PMC11735495; doi:10.1007/s00415-024-12789-0)
Supplement: Supplementary file 5 — Supplementary file5 Supplement E. Coding approach (PDF 37 KB) [file 415_2024_12789_MOESM5_ESM.pdf]

Supplement E. Coding approach

Cycle	Approach	Description	Rating
1	Content coding: symptoms	Spreadsheet coding to identify all reported symptoms of PD at year 2, along with bothersomeness ratings based on position in map.	Patient Reported Symptom Scores (PRSS):<br>• "Most bothersome"=4<br>• "Somewhat bothersome"=3<br>• "Less bothersome"=2<br>• "Not bothersome"=1<br>• "No current issues but still personally important"= 0<br>• "Not relevant or not a symptom of early PD"= 888
2	Content coding: impacts and contributing symptoms	Spreadsheet coding to identify all impacts and symptoms contributing to the impact	• Impacts indicated as *present* (+) or *not present* (.)<br>• Contributing symptoms indicated as contributing (+) or not contributing (.)
3	Content coding: relevance of digital measures<br><br>(maps/pictographs)	Association of digital measures (pictograph) to personally important symptoms and impacts delineated in the map	• Association with most bothersome symptom = 4<br>• Association with somewhat bothersome symptom = 3<br>• Association with less bothersome symptom = 2<br>• Association with symptom that is present but not bothersome = 1<br>• Association with symptom that is not present but important = 0<br>• Perceived as not relevant or not capturing a personal symptom of early PD = 888
4	Content coding: relevance of digital measures<br><br>(interview transcripts)	Standardized content validity questions assessing perceived relevance of digital measures to personally important symptoms	• "Yes" = perceived as relevant to personally important symptoms<br>• "No" = perceived as not important to personally important symptoms<br>• "Qualified" = ambiguous answer (e.g., yes if certain conditions are met, otherwise no)
