# Supplementary material for: Longitudinal qualitative assessment of meaningful symptoms and relevance of WATCH-PD digital measures for people with early Parkinson’s
Source: J Neurol. 2025 Jan 15;272(2):114. doi: 10.1007/s00415-024-12789-0 (PMC11735495; doi:10.1007/s00415-024-12789-0)
Supplement: Supplementary file 6 — Supplementary file6 Supplement F. Supporting data for relevance ratings of WATCH-PD digital measures (PDF 70 KB) [file 415_2024_12789_MOESM6_ESM.pdf]

Supplement F. Supporting data for relevance ratings of WATCH-PD measures

Task	*Minimum*	*Maximum*	*Mean*	*SD*
**The symptom measured is personally meaningful**				
Walking task	7	10	9.5	0.94
Balance task	6	10	9.6	0.86
Tremor task	6	10	9.3	1.2
Finger tapping task	8	10	9.7	0.6
Shape rotation task	7	10	9.6	0.7
Articulation task	5	10	9.2	1.4
Reading task	6	10	9.3	1.1
Phonation task	0	10	8.2	2.3
Visual symbol swap	8	10	9.5	0.8
Trails A & B	0	10	9.3	1.9
**The symptom is currently limiting ability to do things**				
Walking task	0	10	1.9	2.9
Balance task	0	7.5	1.6	2.2
Tremor task	0	10	2.3	2.8
Finger tapping task	0	10	4.3	3.6
Shape rotation task	0	10	4.1	3.6
Articulation task	0	6.5	1.3	2.2
Reading task	0	10	1.9	2.8
Phonation task	0	10	2.1	3.2
Visual symbol swap	0	8.0	1.6	2.8
Trails A & B	0	8	1.2	2.3
**The symptom is currently bothersome.**				
Walking task	0	10	3.3	3.7
Balance task	0	9.0	2.1	2.6
Tremor task	0	10	5.0	3.5
Finger tapping task	0	10	5.4	3.9
Shape rotation task	0	10	5.4	3.9
Articulation task	0	9	2.1	3.1
Reading task	0	10	2.5	3.6
Phonation task	0	10	2.5	3.4
Visual symbol swap	0	10	2.7	3.9
Trails A & B	0	10	1.9	3.6
**The task is a good way to measure the symptom.**				
Walking task	0	10	8.3	2.3
Balance task	0	10	7.2	2.5
Tremor task	0	10	8.7	2.2
Finger tapping task	0	10	8.6	2.5
Shape rotation task	0	10	8.6	2.5
Articulation task	0	10	8.1	2.5
Reading task	0	10	8.8	2.3
Phonation task	0	10	8.1	3.1
Visual symbol swap	0	10	8.4	3.0
Trails A & B	0	10	7.5	2.9

Task	*Minimum*	*Maximum*	*Mean*	*SD*
**The task is similar or relevant to things in real life.**				
Walking task	2	10	8.3	2.1
Balance task	0	10.0	7.3	3.1
Tremor task	0	10.0	5.7	3.8
Finger tapping task	0	10.0	5.5	4.1
Shape rotation task	0	10.0	7.1	3.2
Articulation task	0	10	6.3	3.8
Reading task	0	10.0	8.2	2.8
Phonation task	0	10	6.4	3.7
Visual symbol swap	0	10	8.2	2.2
Trails A & B	0	10	6.1	3.5
**The task is good way to measuring progression of PD symptoms.**				
Walking task	0	10.0	8.9	2.2
Balance task	5	10.0	8.7	1.6
Tremor task	0	10	8.6	2.3
Finger tapping task	0	10.0	8.5	2.9
Shape rotation task	0	10.0	8.3	2.4
Articulation task	0	10	8.0	2.8
Reading task	2	10	8.9	1.9
Phonation task	0	10	8.2	2.8
Visual symbol swap	6	10	9.2	1.2
Trails A & B	0	10.0	8.2	2.6
**The task is personally relevant now (at this stage in disease).**				
Walking task	0	10	7.0	3.5
Balance task	0	10.0	5.7	3.9
Tremor task	0	10	6.5	3.6
Finger tapping task	0	10	7.2	3.7
Shape rotation task	0	10.0	7.2	3.3
Articulation task	0	10	5.7	4.1
Reading task	0	10.0	6.6	4.1
Phonation task	0	10	6.5	4.1
Visual symbol swap	0	10	8.0	3.0
Trails A & B	0	10.0	6.5	3.7
