# Supplementary material for: Longitudinal qualitative assessment of meaningful symptoms and relevance of WATCH-PD digital measures for people with early Parkinson’s
Source: J Neurol. 2025 Jan 15;272(2):114. doi: 10.1007/s00415-024-12789-0 (PMC11735495; doi:10.1007/s00415-024-12789-0)
Supplement: Supplementary file 7 — Supplementary file7 Supplement G. All symptom frequencies. (PDF 132 KB) [file 415_2024_12789_MOESM7_ESM.pdf]

Year 1 SYMPTOM FREQUENCIES

	Not present	Present but not bothersome	Present and a little bothersome	Present and somewhat bothersome	Present and most bothersome
	Row Valid N %	Row Valid N %	Row Valid N %	Row Valid N %	Row Valid N %
Year 1 - Taking PD Medication	57.6%	42.4%	-	-	-
**SENSORY**					
Year 1 - altered sense of taste	100.0%	-	-	-	-
Year 1 - numbness and tingling	100.0%	-	-	-	-
Year 1 - Heachaches	100.0%	-	-	-	-
Year 1 - Loss of appetite	97.0%	-	-	3.0%	-
Year 1 - Double vision/vision changes	97.0%	-	3.0%	-	-
Year 1 - Diminished sensation	97.0%	-	-	-	3.0%
Year 1 - Diminished sense of smell	81.8%	-	9.1%	6.1%	3.0%
Year 1 - Increased pain	60.6%	3.0%	-	6.1%	30.3%
**ANS GU GI**					
Year 1 - Tearing of eyes	97.0%	-	3.0%	-	-
Year 1 - dry eyes	100.0%	-	-	-	-
Year 1 - lower leg swelling	100.0%	-	-	-	-
Year 1 - OTHER urinary problems (inc void, UTI)	97.0%	-	-	-	3.0%
Year 1 - urinary incontinence	97.0%	-	-	-	3.0%
Year 1 - GI Problems	100.0%	-	-	-	-
Year 1 - Blood pressure issues	100.0%	-	-	-	-
Year 1 - Temperature dysregulation	97.0%	-	-	-	3.0%
Year 1 - Feeling Dizzy/lightheaded	93.9%	-	3.0%	-	3.0%
Year 1 - Impotence (sexual dysfunction)	97.0%	-	-	-	3.0%
Year 1 - Urinary frequency/urgency	63.6%	6.1%	6.1%	9.1%	15.2%
Year 1 - Wake up to go to BR	30.3%	12.1%	12.1%	12.1%	33.3%
Year 1 - Constipation	69.7%	6.1%	3.0%	6.1%	15.2%
**COGNITIVE**					
Year 1 - trouble multitasking/processing	81.8%	-	3.0%	3.0%	12.1%
Year 1 - Visual spatial depth perception	69.7%	9.1%	6.1%	9.1%	6.1%
Y1_Left/Right confusion	97.0%	-	-	3.0%	-
Year 1 - Word findings issues	54.5%	3.0%	6.1%	15.2%	21.2%
Year 1 - Difficulty remembering	45.5%	-	18.2%	18.2%	18.2%
Year 1 - Slower thinking	42.4%	-	21.2%	12.1%	24.2%
Year 1 - Difficulty concentrating	33.3%	3.0%	24.2%	21.2%	18.2%
Year 1 - Any cognitive issues	18.2%	3.0%	30.3%	24.2%	24.2%
SLEEP					

Year 1 - Acting out dreams/vivid dreaming	69.7%	3.0%	3.0%	12.1%	12.1%
Year 1 - Daytime sleepiness	63.6%	3.0%	3.0%	9.1%	21.2%
Year 1 - Tired or fatigued	33.3%	6.1%	12.1%	15.2%	33.3%
Year 1 - Insomnia/ interrupted sleep	36.4%	6.1%	15.2%	12.1%	30.3%
**MOOD/PSYCH**					
Year 1 - Flat mood	97.0%	-	-	-	3.0%
Year 1 - Stress	93.9%	-	3.0%	-	3.0%
Year 1 - Personality changes	100.0%	-	-	-	-
Year 1 - Irritability/frustration	90.9%	-	6.1%	-	3.0%
Year 1 - More emotional	97.0%	-	3.0%	-	-
Year 1 - Apathy	87.9%	-	3.0%	3.0%	6.1%
Year 1 - Depression/Sadness	57.6%	3.0%	15.2%	15.2%	9.1%
Year 1 - Anxiety	48.5%	-	18.2%	15.2%	18.2%
VOICE AND SWALLOW					
Year 1 - Throat clearing	100.0%	-	-	-	-
Year 1 - Dry mouth	97.0%	-	3.0%	-	-
Year 1 - Hypersalivation/drooling	84.8%	3.0%	6.1%	6.1%	-
Year 1 - Swallowing/Choking	84.8%	-	-	6.1%	9.1%
Year 1 - Change in vocal quality	72.7%	-	6.1%	9.1%	12.1%
Year 1 - Monotone voice	72.7%	6.1%	12.1%	-	9.1%
Year 1 - ArticulatIon	54.5%	9.1%	15.2%	12.1%	9.1%
Year 1 - Quiet voice	36.4%	12.1%	24.2%	12.1%	15.2%
**GROSS MOTOR**					
Year 1 - Twitching	97.0%	-	3.0%	-	-
Year 1 - Tripping and falling	78.8%	3.0%	9.1%	-	9.1%
Year 1 - Restless leg	97.0%	-	3.0%	-	-
Year 1 - Freezing	100.0%	-	-	-	-
Year 1 - General incoordination/motor control	72.7%	3.0%	-	6.1%	18.2%
Year 1 - Dyskinesias	100.0%	-	-	-	-
Year 1 - Altered facial expression	87.9%	3.0%	3.0%	-	6.1%
Year 1 - Muscle fatigue	78.8%	-	-	3.0%	18.2%
Year 1 - Muscle weakness	72.7%	3.0%	-	12.1%	12.1%
Year 1 - Decreased Range of Motion	63.6%	3.0%	3.0%	12.1%	18.2%
Year 1 - Postural issues	87.9%	3.0%	3.0%	-	6.1%
Year 1 - Altered arm swing	63.6%	6.1%	3.0%	9.1%	18.2%
Year 1 -Balance issues	33.3%	18.2%	15.2%	12.1%	21.2%
Year 1 - Gait difficulties/changes	39.4%	15.2%	3.0%	12.1%	30.3%
Year 1 - Slow movements	18.2%	21.2%	3.0%	15.2%	42.4%
Year 1 - Spasms and cramping/dystonia	66.7%	3.0%	9.1%	3.0%	18.2%
Year 1 - Stiffness, Rigidity	54.5%	3.0%	3.0%	6.1%	33.3%

**FINE MOTOR**					
Year 1 - Trouble Grooming or Getting dressed	57.6%	3.0%	3.0%	9.1%	27.3%
Year 1 trouble with Finger coordination	42.4%	3.0%	3.0%	6.1%	45.5%
Year 1 - trouble Using Tools/Utensils	33.3%	3.0%	6.1%	15.2%	42.4%
Year 1 - Trouble using a keyboard or mouse	30.3%	9.1%	3.0%	12.1%	45.5%
Year 1 - fine motor dexterity problems	27.3%	9.1%	-	12.1%	51.5%
**TREMOR**					
Year 1 - sense of internal tremor	93.9%	-	6.1%	-	-
Year 1 - face or jaw tremor	87.9%	-	3.0%	-	9.1%
Year 1 - leg/foot tremor	57.6%	3.0%	12.1%	-	27.3%
Year 1 - hand tremor	18.2%	3.0%	15.2%	9.1%	54.5%
Year 1 - Any Tremor	6.1%	3.0%	15.2%	9.1%	66.7%

Year 2 SYMPTOM FREQUENCIES

	Not present	Present but not bothersome	Present and a little bothersome	Present and somewhat bothersome	Present and most bothersome
	Row Valid N %	Row Valid N %	Row Valid N %	Row Valid N %	Row Valid N %
Year 2 - Taking PD Medication	42.4%	57.6%	-	-	-
**SENSORY**					
Year 2 - altered sense of taste	90.9%	3.0%	3.0%	3.0%	-
Year 2 - numbness and tingling	93.9%	-	3.0%	-	3.0%
Year 2 - headaches	97.0%	-	-	3.0%	-
Year 2 - Loss of appetite	97.0%	-	3.0%	-	-
Year 2 - Double vision/vision changes	97.0%	-	-	3.0%	-
Year 2 - Diminished sensation	84.8%	3.0%	6.1%	3.0%	3.0%
Year 2 - Diminished sense of smell	54.5%	12.1%	15.2%	18.2%	-
Year 2 - Increased pain	48.5%	9.1%	12.1%	3.0%	27.3%
**ANS GU GI**					
Year 2 - Tearing of eyes	97.0%	-	3.0%	-	-
Year 2 - dry eyes	97.0%	-	-	-	3.0%
Year 2 - lower leg swelling	97.0%	3.0%	-	-	-
Year 2 - OTHER urinary problems (inc void, UTI)	93.9%	-	-	-	6.1%
Year 2 - urinary incontinence	87.9%	-	-	3.0%	9.1%
Year 2 - GI Problems	93.9%	3.0%	-	-	3.0%
Year 2 - Blood pressure issues	93.9%	-	-	6.1%	-
Year 2 - Temperature dysregulation	100.0%	-	-	-	-
Year 2 - Feeling Dizzy/lightheaded	78.8%	6.1%	3.0%	6.1%	6.1%
Year 2 - Impotence (sexual dysfunction)	97.0%	-	-	3.0%	-
Year 2 - Urinary frequency/urgency	51.5%	3.0%	9.1%	18.2%	18.2%
Year 2 - Wake up to go to BR	51.5%	12.1%	6.1%	9.1%	21.2%
Year 2 - Constipation	48.5%	18.2%	9.1%	9.1%	15.2%
**COGNITIVE**					
Year 2 - trouble multitasking/processing	57.6%	-	9.1%	9.1%	24.2%
Year 2 - Visual spatial depth perception	54.5%	9.1%	15.2%	15.2%	6.1%
Year 2 - Left/Right confusion	100.0%	-	-	-	-
Year 2 - Word findings issues	39.4%	3.0%	12.1%	24.2%	21.2%
Year 2 - Difficulty remembering	24.2%	12.1%	12.1%	21.2%	30.3%
Year 2 - Slower thinking	33.3%	3.0%	15.2%	21.2%	27.3%
Year 2 - Difficulty concentrating	42.4%	3.0%	12.1%	15.2%	27.3%

Year 2 - Any cognitive issues	12.1%	9.1%	15.2%	30.3%	33.3%
**SLEEP**					
Year 2 - Acting out dreams/vivid dreaming	72.7%	6.1%	3.0%	6.1%	12.1%
Year 2 - Daytime sleepiness	69.7%	3.0%	-	9.1%	18.2%
Year 2 - Tired or fatigued	24.2%	9.1%	18.2%	15.2%	33.3%
Year 2 - Insomnia/ interrupted sleep	27.3%	24.2%	9.1%	9.1%	30.3%
**MOOD/PSYCH**					
Year 2 - Flat mood	97.0%	-	3.0%	-	-
Year 2 - Psychosis	97.0%	-	-	-	3.0%
Year 2 - Stress	90.9%	-	-	3.0%	6.1%
Year 2 - Personality changes	75.8%	-	9.1%	15.2%	-
Year 2 - Irritability/frustration	72.7%	3.0%	9.1%	9.1%	6.1%
Year 2 - More emotional	90.9%	-	-	-	9.1%
Year 2 - Apathy	69.7%	6.1%	9.1%	15.2%	-
Year 2 - Depression/Sadness	69.7%	9.1%	6.1%	12.1%	3.0%
Year 2 - Anxiety	51.5%	15.2%	9.1%	12.1%	12.1%
Year 2 – General mood changes	27.3%	15.2%	18.2%	30.3%	9.1%
**VOICE AND SWALLOWING**					
Year 2 - Throat clearing	97.0%	3.0%	-	-	-
Year 2 - Dry mouth	90.9%	-	3.0%	6.1%	-
Year 2 - Hypersalivation/drooling	51.5%	27.3%	12.1%	6.1%	3.0%
Year 2 - Swallowing/Choking	54.5%	9.1%	15.2%	9.1%	12.1%
Year 2 - Change in vocal quality	42.4%	6.1%	15.2%	21.2%	15.2%
Year 2 - Monotone voice	60.6%	6.1%	12.1%	9.1%	12.1%
Year 2 - Articulation	51.5%	9.1%	9.1%	18.2%	12.1%
Year 2 - Quiet voice	30.3%	15.2%	18.2%	21.2%	15.2%
**GROSS MOTOR**					
Year 2 - Twitching	93.9%	-	-	-	6.1%
Year 2 - Tripping and falling	75.8%	-	9.1%	-	15.2%
Year 2 - RLS	90.9%	-	-	3.0%	6.1%
Year 2 - Freezing	97.0%	-	-	-	3.0%
Year 2 - General incoordination/motor control	60.6%	-	3.0%	6.1%	30.3%
Year 2 - Dyskinesias	84.8%	-	-	-	15.2%
Year 2 - Altered facial expression	63.6%	15.2%	18.2%	3.0%	-
Year 2 - Muscle fatigue	78.8%	-	-	3.0%	18.2%
Year 2 - Muscle weakness	66.7%	3.0%	3.0%	3.0%	24.2%
Year 2 - Decreased Range of Motion	75.8%	-	6.1%	3.0%	15.2%
Year 2 - Postural issues	54.5%	18.2%	9.1%	12.1%	6.1%

Year 2 - Altered arm swing	60.6%	15.2%	9.1%	6.1%	9.1%
Year 2 - Balance issues	24.2%	18.2%	12.1%	24.2%	21.2%
Year 2 - Gait difficulties/changes	15.2%	12.1%	21.2%	15.2%	36.4%
Year 2 - Slow movements	9.1%	9.1%	27.3%	15.2%	39.4%
Year 2 - Spasms and cramping/dystonia	60.6%	12.1%	6.1%	9.1%	12.1%
Year 2 - Stiffness, Rigidity	27.3%	15.2%	12.1%	6.1%	39.4%
**FINE MOTOR**					
Year 2 - Grooming & Getting dressed	51.5%	-	6.1%	15.2%	27.3%
Year 2 - Finger coordination	42.4%	-	9.1%	15.2%	33.3%
Year 2 - Using Tools/Utensils	30.3%	3.0%	12.1%	15.2%	39.4%
Year 2 - Altered keyboarding/mousing/phone	27.3%	3.0%	12.1%	18.2%	39.4%
Year 2 - Altered hand writing	24.2%	12.1%	18.2%	6.1%	39.4%
**TREMOR**					
Year 2 - Sense of internal tremor	93.9%	-	-	3.0%	3.0%
Year 2 - Other (Jaw, neck, face) tremor	84.8%	3.0%	-	6.1%	6.1%
Year 2 - Leg/foot tremor	63.6%	-	9.1%	3.0%	24.2%
Year 2 - Hand/arm tremor	18.2%	3.0%	12.1%	24.2%	42.4%
Year 2 – Any tremor	3.0%	3.0%	15.2%	30.3%	48.5%
